# The demonstration of significant ferroelectricity in epitaxial Y-doped HfO_2_ film

**DOI:** 10.1038/srep32931

**Published:** 2016-09-09

**Authors:** Takao Shimizu, Kiliha Katayama, Takanori Kiguchi, Akihiro Akama, Toyohiko J. Konno, Osami Sakata, Hiroshi Funakubo

**Affiliations:** 1 Materials Research Center for Element Strategy, Tokyo Institute of Technology, 4259 Nagatsuta, Midori, Yokohama 226-8503, Japan; 2 Interdisciplinary Graduate School of Science and Engineering, Tokyo Institute of Technology, 4259 Nagatsuta, Midori, Yokohama 226-8502, Japan; 3 Institute for Materials Research, Tohoku University, 2-1-1 Katahira, Aoba-ku, Sendai 980-8577, Japan; 4 Synchrotron X-ray Station at SPring-8, National Institute for Materials Science, 1-1-1 Koto, Sayo, Hyogo 679-5148, Japan; 5 School of Materials and Chemical Technology, Tokyo Institute of Technology, 4259 Nagatsuta, Midori, Yokohama 226-8502, Japan

## Abstract

Ferroelectricity and Curie temperature are demonstrated for epitaxial Y-doped HfO_2_ film grown on (110) yttrium oxide-stabilized zirconium oxide (YSZ) single crystal using Sn-doped In_2_O_3_ (ITO) as bottom electrodes. The XRD measurements for epitaxial film enabled us to investigate its detailed crystal structure including orientations of the film. The ferroelectricity was confirmed by electric displacement filed – electric filed hysteresis measurement, which revealed saturated polarization of 16 μC/cm^2^. Estimated spontaneous polarization based on the obtained saturation polarization and the crystal structure analysis was 45 μC/cm^2^. This value is the first experimental estimations of the spontaneous polarization and is in good agreement with the theoretical value from first principle calculation. Curie temperature was also estimated to be about 450 °C. This study strongly suggests that the HfO_2_-based materials are promising for various ferroelectric applications because of their comparable ferroelectric properties including polarization and Curie temperature to conventional ferroelectric materials together with the reported excellent scalability in thickness and compatibility with practical manufacturing processes.

Ferroelectric materials with switchable spontaneous polarization by an electric field are considered to be essential for next-generation nonvolatile memories, sensors, actuators, and electro-optic devices^1^,^2^,^3^,^4^,^5^,^6^. Over several decades, new ferroelectric materials have been widely investigated in order to produce devices with high performance. In particular, materials with the perovskite and related structure have garnered enormous interest because of their chemical and thermal robustness and their other attractive characteristics including high dielectric constant, large spontaneous polarization, and electro-mechanical response^2^. Despite these excellent characteristics, perovskite materials still pose the difficult problem of the size effect: as these materials are scaled down in thickness, their ferroelectric properties degrade, making them difficult to integrate into devices^7^,^8^. Furthermore, perovskite ferroelectrics are not especially compatible with silicon-based semiconductor device technology^9^,^10^. For example, during post-metallization annealing under an H_2_ atmosphere, which is essential for Si-based devices, H^+^ is incorporated into the perovskite ferroelectrics, hampering the device’s performance^9^,^10^.

Recently, a new class of ferroelectrics has been discovered, HfO_2_-based ferroelectrics, which might open a new path to solving the problems associated with scaling down^11^,^12^. These materials have exhibited ferroelectricity without degradation down to a thickness of 5 nm, even in polycrystalline films, making them some of the most promising candidates for materials that avoid the size effect. These HfO_2_-based materials are a key dielectric material, as high-*k* gate insulators are in current Si-based semiconductor technology^13^,^14^. These materials promise high compatibility with Si-based CMOS technology, which would enable highly integrated ferroelectric devices and energy-related capacitor applications^15^,^16^. Indeed, a ferroelectric field-effect transistor has already been demonstrated with a gate length of 28 nm using 9-nm-thick HfO_2_-based films^17^,^18^.

The ferroelectricity in HfO_2_-based materials seems to originate from the metastable orthorhombic structure belonging to the *Pca*2_1_ space group, which was first proposed in studying the orthorhombic structure of Mg-doped ZrO_2_ with neutron diffraction^11^,^19^,^20^. Several pioneering works on formation of orthorhombic phase in HfO_2_ films have been performed^21^,^22^. ZrO_2_, HfO_2_, and related materials exist in various crystal structures, including cubic, tetragonal, and monoclinic symmetries. However, these structures have an inversion center, which prevents ferroelectricity^23^. In contrast, the proposed orthorhombic phase has a noncentrosymmetric and polar structure, so it might exhibit ferroelectricity. In studies on ferroelectric HfO_2_-based materials, X-ray diffraction (XRD) has given indirect evidence for the orthorhombic structure with *Pca*2_1_ because centrosymmetric orthorhombic phases belonging to *Pbca* and *Pbcm* are also known for ZrO_2_ and HfO_2_^11^,^24^,^25^,^26^,^27^,^28^,^29^. Because the anions in these materials are arranged quite similarly to the *Pca*2_1_ phase, it is difficult to distinguish them by XRD especially for thin films. Recently, transmission electron microscopy (TEM), scanning TEM (STEM), and convergent electron beam diffraction have shown that the ferroelectric phase in HfO_2_-based materials has a polar structure^28^. After this study, we grew a single-crystal epitaxial HfO_2_-based films with a single polar orthorhombic phase by pulsed laser deposition (PLD)^30^. In the previous study, we probed the polar crystal structure by XRD and annular bright-field STEM that visualized the atomic arrangement, including light O atoms^30^.

In numerous studies of conventional ferroelectrics, epitaxial structures have enabled great successes in investigating their nature^8^,^31^,^32^,^33^. Epitaxial films can have interfaces that are well defined at the atomic scale, which allows us to clarify these films’ ferroelectricity, including charge screening and depolarization field, which are essential in ferroelectric thin films with thicknesses of nanometers, producing new functionality such as giant resistive switching in ferroelectric tunneling junctions^34^,^35^. Even more, epitaxial films can be used to study fundamental electrical and structure properties such as spontaneous polarization and domain structure^31^,^36^. However, almost all reports on the ferroelectricity of HfO_*2*_-based materials had used polycrystalline films^11^,^25^,^37^,^38^,^39^, which makes difficult to estimate the fundamental information of novel categories of HfO_2_-based ferroelectrics, including the spontaneous polarization and the Curie temperature. Furthermore, it is difficult to evaluate the performance of these films to the real device applications. Hence, demonstrating ferroelectricity using epitaxial HfO_2_-based films might be crucial in exploring their properties and using them in practical applications.

Recently, we grew an epitaxial orthorhombic YO_1.5_-substituted HfO_2_ film, which gives us the opportunity to study these films’ ferroelectricity from the perspective of crystal structure^30^. However, an appropriate bottom electrode has not yet been developed for HfO_2_-based films, hindering their ferroelectric polarization switching. Even more, the electrode acts as a buffer layer for epitaxial growth of the HfO_2_-based films. In this study, we demonstrate ferroelectricity in 0.07YO_1.5_–0.93 HfO_2_ (YHO-7) film by using Sn-doped In_2_O_3_ (ITO) bottom electrode grown on (110) yttrium oxide -stabilized zirconium oxide (YSZ) substrate. The film shows well-defined electric displacement filed – electric filed (***D*****–*****E***) hysteresis loops with saturation polarization of ~16 μC/cm^2^. In addition, structural phase transition from orthorhombic to tetragonal phases is confirmed by high temperature X-ray diffraction.

## Results

**Figure 1:**
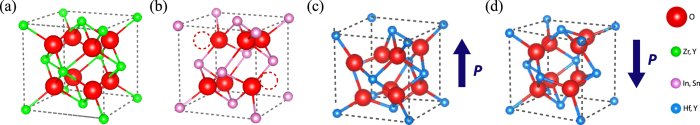
Schematic crystal structures of (**a**) YSZ (cubic fluorite), (**b**) ITO (bixbyite), and orthorhombic phase of YHO with (**c**) upward and (**d**) downward spontaneous polarization^52^. For ease of comparison, only one-eighth of the ITO unit cell is depicted. The open, dashed circles indicate unoccupied oxygen sites.

To obtain material’s ferroelectric properties, a metal–ferroelectric–metal capacitor heterostructure is needed. The bottom electrode is essential because it acts as a bottom electrode for electrical measurements and as a substrate for epitaxial growth. Thus, the bottom electrode must have similar lattice parameters and, desirably, crystal structure with YSZ (see Fig. 1(a)) and also YHO-7 (Fig. 1(c,d)) together with good conductivity. We tried to insert the conductive ITO layers between YHO-7 and YSZ single crystal having fluorite structure. ITO has a bixbyite structure as illustrated in Fig. 1(b) and is often used as an oxide electrode because of its good conductivity and excellent optical transparency. Here, we focus on the crystal structure of ITO, the bixbyite structure. The bixbyite structure is a deficient fluorite structure, so we expect its lattice parameters and atomic configuration to be similar to those of YSZ and HfO_2_. In reality, ITO has slightly smaller lattice parameters than both the YSZ substrate and the HfO_2_-based dielectric layer, but it remains a feasible material for the bottom electrode. However, the previous study on the epitaxial growth on (100)ITO/(100)YSZ shows that the epitaxial YHO-7 film on the substrate tend to orient non-polar longer *a*-axis^40^. To obtain the polarization along out-of-plane component, we attempt to grow YHO-7 film on (110) ITO/(110)YSZ substrate in the present study.

**Figure 2:**
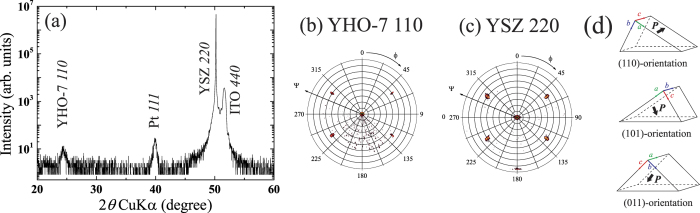
(**a**) *θ*–2*θ* XRD patterns measured from 20 to 60° for a Pt/YHO-7/ITO/YSZ heterostructure. Also shown are pole figures for (**b**) YSZ 220 diffraction at 2*θ* of 50.1° and (**c**) orthorhombic YHO-7 110 diffraction at 2*θ* of 24.6°. (**d**) Three expected crystal orientations for YHO-7 film on ITO//(110)YSZ substrate. The polarization domain was illustrated for each orientation.

Figure 2(a) shows the *θ*–2*θ* XRD pattern for the Pt/YHO-7/ITO//(110)YSZ capacitor. The 7% YO_1.5_ doping concentration was chosen according to the previous studies^30^,^40^. This concentration is optimized value within our studies. This value is slightly higher than the reported values for the films prepared by other deposition techniques. The deferent optimal value might stem from the different deposition techniques because these studies show also different optimal doping concentrations^25^,^41^,^42^. In particular, direct crystallization employed in the present study instead of post crystallization by heat treatment probably gives different doping concentration. The peaks from YSZ, ITO, and Pt—indexed as 220, 440, and 111, respectively—appear in this pattern, suggesting an epitaxial growth of ITO bottom electrode. Only a weak 110 peak from the YHO-7 film appeared in this *θ*–2*θ* XRD pattern, which is characteristic of its orthorhombic phase. Note that 101 and 011 diffraction is forbidden by the extinction rule for *Pca*2_1_ orthorhombic phase. On the other hand, {220} diffraction—220, 202, and 022—from YHO-7 did not appear. This result may have occurred because of the significant overlapping between the 220/202 peaks from the YHO-7 film and the 220 peak from the YSZ substrate. According to our previous study, the lattice parameters of *b*- and *c*-axes of the *Pca*2_1_ orthorhombic phase, which are quite similar value of around 5.08 Å, are smaller than that of the YSZ substrate (5.145 Å), while the lattice parameter of the *a*-axis (5.22 Å) is larger than those of the *b*- and *c*-axes and the YSZ substrate^25^. These facts mean that the (220) and (202) lattice spacings of YHO-7 are calculated to be close to (220) lattice spacing of YSZ, leading to peak overlaps and implying that (110) and/or (101)-oriented YHO-7 is grown on the substrate. The 110 peak from the YHO-7 film in Fig. 2(a) also supports the existence of the (110)-oriented domain in the YHO-7 film. The lattice spacing of the (220) plane of the YHO-7 film, which is half of lattice spacing of the (110) plane, calculated from the 110 peak, is ~3.645 Å. This spacing is very close to that of the (220) plane of the YSZ substrate, so it likely obscures the 220 peaks of the YHO-7 film. Similarly 202 peaks also cannot appear due to the close lengths of *b*- and *c*-axes of YHO-7 if there is (101)-orientation. It should be noted that the lack of the 101 peak does not mean the absence of (101)-oriented domain because the 101 peak does not diffract from a film with the *Pca*2_1_ structure.

To confirm the epitaxial growth of the YHO-7 layer, we measured pole figures. Figure 2(b) shows the X-ray pole figure measured with respect to the inclination (*Ψ*) and azimuth (*ϕ*) angles for the 110 peak of the YHO-7 film, and Fig. 2(c) shows the same for the 220 peak of the YSZ substrate. The 110 peak is a superlattice peak accompanied by lowering of the crystal symmetry, indicating that the YHO-7 film is orthorhombic. On the other hand, no 110 diffraction peak appears for the films with a cubic or tetragonal fluorite structure, such as the YSZ substrate. The 110 peak from the YHO-7 and the 220 peak from the YSZ appear to be located at similar *Ψ* and *ϕ* angles as shown in Fig. 2(b,c), indicating the epitaxial growth of the YHO-7 film on the ITO-buffered YSZ substrate. It is worth mentioning that here we use the “epitaxial film” for the film possessing well-defined relation with substrate for both in-plane and out-of-plane directions. This term can be found to be used in the same manner for other ferroelectric thin films even if they have multi-domain structures at room temperature^36^,^43^. Note that the films should grow on the substrate with single domain epitaxially at the deposition temperature because of their tetragonal symmetry at the growth temperature.

The 110 diffraction helps us to consider the domain structure of the YHO-7 film. Assuming that an orthorhombic YHO-7 film is present on the (110) YSZ substrate, we expect three possible domains with orientations of (110), (011), and (101) as shown in Fig. 2(d). The (110)-oriented domain appears to exist in the film, as evidenced by the appearance of *110* diffraction, as shown in Fig. 1(a), where the scattering vectors of the diffracted peak are parallel to the surface normal direction. In contrast, the (011)-oriented domain can be excluded because there is no *022* peak on *θ*–2*θ* XRD pattern shown in Fig. 2(a), that must appear due to the significant difference in lattice spacing between the (022) plane of the YHO-7 film and the (220) plane of the YSZ substrate. The 110 peak observed at *Ψ* of around 60° in Fig. 2(b) suggests the existence of the (011)-, and/or (101)-oriented domains, which have (110) plane tilting by ~60° with respect to the surface normal. This means that the presence of the (101)-oriented domains as (110)-oriented one is ruled out by above consideration.

The XRD results suggest that the YHO-7 film include both the (110)- and (101)-oriented domains. These domains may have formed because of a phase transition at the Curie temperature (*T*_C_) during the cooling process after the film deposition. A previous study revealed an orthorhombic–tetragonal phase transition occurs at ~450 °C^30^. The longest *a*-axis in the orthorhombic phase should transform to the longer *c*-axis in the tetragonal phase, while the shorter *b*- and *c*- axes in the orthorhombic phase should change diagonal of the square consisting of the shorter *a*-axis in the tetragonal phase through the phase transition. Note that the unit cell of the orthorhombic *Pca*2_1_ phase has the double cell volume of that of tetragonal phase. This leads replacement of the axes at the phase transition. The present result was readily understood by the following assumption; the (112)-oriented variant of tetragonal phase with *P*4_2_/*nmc* space group was grown at the deposition temperature and it transformed (110)- and (101)-oriented domains by the structural phase transition.

**Figure 3:**
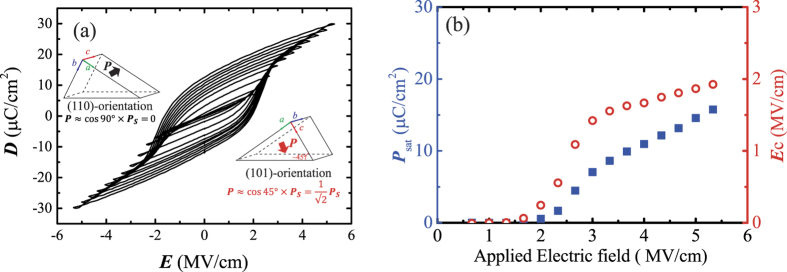
(**a**) ***D–E*** hysteresis curve of Pt/(YHO-7)/ITO capacitor measured with a triangular electric field at 10 kHz. The insets show schematics of the domains included in the present film. (**b**) The saturated polarization (***P***_*sat*_; closed squares) and the coercive field (***E***_**c**_; open circles) as functions of applied field.

Demonstrating polarization switching accompanied by ferroelectricity in the present film is most crucial. Figure 3(a) shows the ***D***–***E*** hysteresis curves measured for the YHO-7 film on the ITO//(110) YSZ substrate. A relatively high 10 kHz triangular electric field was used for the ***D*****–*****E*** hysteresis curve measurement to eliminate the effect from leakage current. The hysteresis curves were recorded after applying 100 cycles. These hysteresis curves had square shapes. The slope of the ***D**–**E*** curve near coercive field is still slight even with the epitaxial film. This might be due to the fact that the present film does not have perfect polar axis orientation. Similar example can be found in the study on Pb(Zr, Ti)O_3_ 44. Figure 3(b) shows the saturated polarization and coercive field as functions of the applied electric field. The coercive field as a function of applied field exhibited well-saturated behavior, indicating normal ferroelectric behavior. Also, at the maximum applied field of 5.3 MV/cm, the saturated polarization (***P***_sat_) reached to be ~16 μC/cm^2^.

## Discussion

The well-defined orientation of the epitaxial film allowed us to evaluate an important physical property: the spontaneous polarization (***P*****s**). The XRD results suggest that the present film contain domains of two orientations: (110) and (101). As schematized in Fig. 2(d), the (110)-oriented domain includes *a*- and *b*-axes as the out-of-plane component, while the (101)-oriented domain includes the *a*- and *c*-axes. Because the orthorhombic phase with the *Pca*2_1_ space group has a polarization direction along the *c*-axis, the (101)-oriented domain is the ferroelectrically active domain when the electric field is applied along the out-of-plane direction.

As the *c*-axis tilts by ~45° with respect to the surface normal direction, the domain should exhibit a polarization of about 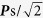. On the other hand, the (110) domain contributes less to the ferroelectricity (see inset schematics of Fig. 3(a)). The volume fraction of the domain in ferroelectric film is generally determined by spontaneous stain accompanied by the ferroelectric phase transition due to the condition of substrate clamping. It have been demonstrated that small spontaneous strain tend to lead nearly even distribution as demonstrated in SrBi_2_Nb_2_O_9_, which has very small spontaneous strain^45^. This is similar the case where spontaneous strain of around 0.2% for orthorhombic HfO_2_. Therefore, it is reasonable to assume that the (110) and (101) domains take up the same volume fraction, because the (110)/(101) domain structure might be generated by the phase transition, and the *b*- and *c*-axes have very similar lattice constants at room temperature. Thus, the strain accompanied with the phase transition and the driving force toward the preferred orientation are small. As a consequence, we assume the observed macroscopic polarization from the entire film to be ~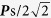.

Combining this assumption and the observed saturated polarization of 16 μC/cm^2^, we estimate that this film has a ***P*****s** of 45 μC/cm. In first-principles theoretical studies, Clima *et al*. calculated***P*****s** for ferroelectric HfO_2_ materials with various dopants, finding values from 40 μC/cm^2^ for Y-doped HfO_2_ to 53 μC/cm^2^ for Gd-HfO_2_ 46. These results agree with the *P***s** estimated here, indicating that the present epitaxial film exhibited intrinsic properties. Our study gives the first experimental evaluation of ***P*****s** that agrees well with theoretical predictions. It should be discussed about possibility that the domain switching induced by the electric field. The slight increase in polarization by an applied field in saturation curve implies that the small increase in polar active (101)-oriented domain. However, generally 90° domain switching requires high electric field compared to 180° domain switching. In actual the little domain switching can be observed for the Nd-substituted Bi_4_Ti_3_O_12_ 43. Note that the further increase in applied electric field might induce larger amount of 90° domain switching, although the present study could not be achieved due to the limitation from electric breakdown.

The experimentally observed polarization in this study is almost the same or smaller than that for previous studies on polycrystalline film^25^,^27^,^28^. This is probably due to the domain configuration of the present film, as mentioned above. Assuming the random orientation for polycrystalline film, we can expect the ***P***_sat_ of ***P*****s**/2. This value is larger than the expected value for the present study, when the domain configuration is taken into account. Another possibility is associated with the non-180° domain switching in polycrystalline film. This switching possibly is stimulated in polycrystalline films due to smaller grain size than epitaxial films. In addition, substitution of cations with larger ionic radii might give large polarization. This tendency can be confirmed in theoretical calculations^46^.

**Figure 4:**
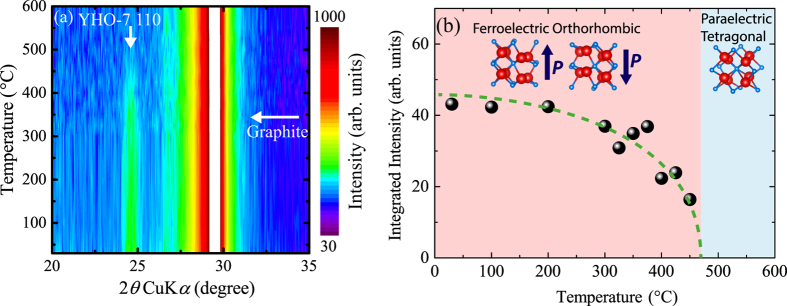
(**a**) Temperature dependence of X-ray diffraction pattern between 20° and 35° from room temperature to 600 °C. The intensity of profiles was displayed according to the color bar. (**b**) Integrated intensity of YHO-7 110 diffraction peak as a function of temperature.

The Curie temperature is also important ferroelectric nature from the both scientific and industrial viewpoints. Figure 4 (a) shows the temperature dependence of the XRD pattern from 20° to 35°. Strong peak appearing at 30° is the diffraction from the graphite dome of the measurement set up, while the weak peak located at 24.5° is 110 diffraction peak from YHO-7, which also can be seen in Fig. 2(a). 110 peak from YHO-7 became weaker with increased in temperature, and disappeared at 475 °C. The integrated intensity of 110 YHO-7 peak is plotted in Fig. 4(b). This figure clearly indicates that the YHO-7 film undergoes the orthorhombic to tetragonal phase transition at around 450 °C. This temperature might correspond to *T*_C_. Note that estimated *T*_C_ in the present study is in accordance with that obtained in previous study for epitaxial YHO-7 film on (100)YSZ substrate without ITO bottom electrode^25^.

**Figure 5:**
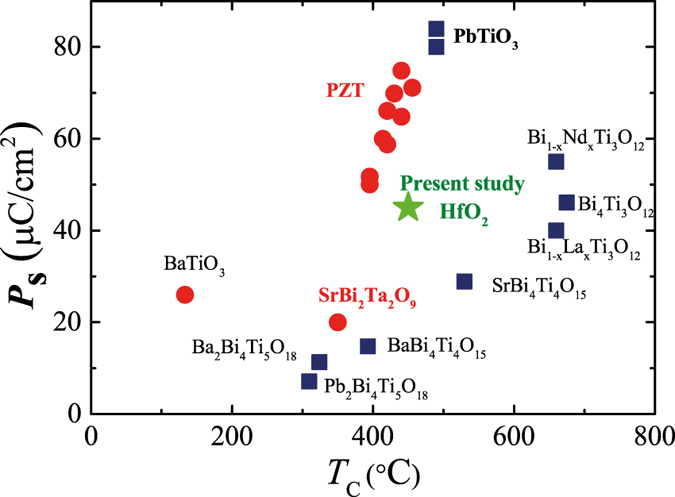
Relationship between the Curie temperature (*T*_C_) and spontaneous polarization (***P***_*sat*_) for conventional ferroelectric materials and the present YHO-7 film. The materials include BaTiO_3_ 47,48, PbTiO_3_ 53, PbZr_1−*x*_Ti_*x*_O_3_ 50,54, SrBi_2_Ta_2_O_9_ 49, Pb_2_Bi_4_Ti_5_O_18_ 55, Ba_2_Bi_4_Ti_5_O_18_ 55, BaBi_4_Ti_4_O_15_ 55, Sr_2_Bi_4_Ti_4_O_15_ 56, Bi_4_Ti_3_O_12_ 57,58, Bi_4−*x*_La_*x*_Ti_3_O_12_ 59,60, and Bi_4−*x*_Nd_*x*_Ti_3_O_12_ 43,61.

It is meaningful to compare the ferroelectric properties of HfO_2_ with those of other ferroelectric materials. Figure 5 shows an estimated***P*****s** of 45 μC/cm^2^ and the *T*_C_ of 450 °C from the present study for the YHO-7 film together with those of well-known materials such as BaTiO_3_ (***P*****s** = 27 μC/cm^2^, *T*_C_ = 130 °C)^47^,^48^, SrBi_2_Ta_2_O_9_ (***P*****s** = 22 μC/cm^2^, *T*_C_ = 355 °C)^49^, and PZT (***P*****s** = ~60 μC/cm^2^, *T*_C_ = ~420 °C; Zr/(Zr + Ti) = 0.4, which is a composition for practical use)^50^. As summarized in Fig. 5, HfO_2_-based materials have the potential to perform as well as other ferroelectric materials. It should be emphasized the HfO_2_-based ferroelectric film has unique feature including the CMOS compatibility and robustness against the miniaturization and hydrogen atmosphere that cannot be obtained by using the other perovskite related materials^17^,^51^. Figure 5 reveals that HfO_2_-based ferroelectric film gives these features as well as the comparable ferroelectric properties. Thus, HfO_2_-based materials are promising candidates for various applications—including ferroelectric random-access memory and future memory technologies such as ferroelectric tunnel junctions and piezoelectric transistors—because of their desirable characteristics such as their high compatibility to silicon-based technology and their ferroelectricity, even in ultra-thin films.

## Conclusion

In summary, we attempted to grow the epitaxial orthorhombic YHO-7 film on ITO//(110)YSZ and demonstrate the ferroelectricity of the epitaxial film for the first time. To characterize their electrical properties, we fabricated an epitaxial ITO bottom electrode on the (110) YSZ substrate, which also enabled us to grow the epitaxial orthorhombic YHO-7 film. XRD measurements revealed the orientation and domain configuration of the YHO-7 film. The YHO-7 film exhibited ferroelectricity with square ***D–E*** hysteresis loops and a saturated polarization of ~16 μC/cm^2^. Estimated spontaneous polarization based on the obtained saturated polarization and the crystal structure analysis was 45 μC/cm^2^, which is in good agreement with theoretical value predicted by first principle calculation. Also Curie temperature was estimated to be about 450 °C from high temperature XRD measurement. This result strongly suggests that the epitaxial growth of the film allows to assess the intrinsic physical properties of the orthorhombic ferroelectric HfO_2_-based materials. This study reveals that the HfO_2_-based materials are promising for various ferroelectric applications because of their comparable ferroelectric properties including polarization and Curie temperature to conventional ferroelectric materials.

## Methods

### Sample preparation

We grew 15-nm-thick YHO-7 and 30-nm-thick ITO films on a (110) YSZ single-crystal substrate by PLD with a KrF excimer laser at a wavelength and fluence of 248 nm and 3 J/cm^2^, respectively. For the YHO-7 deposition, a ceramic target was prepared from HfO_2_ and Y_2_O_3_ powders by a conventional solid-state reaction, while the ITO was deposited with a commercial ceramic target (Kojundo Co). The substrate temperature during deposition was kept at 700 °C, while pressures were maintained at 10 and 1 mTorr O_2_ for growth of YHO-7 and ITO, respectively.

### Structural Characterization

The *θ–*2*θ* XRD patterns were measured by a high-resolution diffractometer (SmartLab, Rigaku) with Cu *Kα*_*1*_ radiation (λ = 1.5406 Å) monochromated by channel-cut Ge crystals. The pole figures were measured with a four-axis diffractometer (X’Pert PRO MRD, Panalytical). The high-temperature XRD measurement was performed by D8 Discover (Bruker) with a larger-area two-dimensional detector (VÅNTEC-500, Bruker) and a hot stage (DHS 1100, Anton Paar) with Cu Kα radiation (λ = 1.5418 Å).

### Electrical measurement

The electrical properties of the YHO-7 films were investigated with a ferroelectric tester (FCE, Toyo Corp.), which applied a triangular-wave electric field of 10 kHz and measured the films ***D–E*** hysteresis loops. 100-nm thick Pt electrodes with 100 μm in diameter was prepared by electron beam evaporation method.

## Additional Information

**How to cite this article**: Shimizu, T. *et al*. The demonstration of significant ferroelectricity in epitaxial Y-doped HfO_2_ film. *Sci. Rep.* **6**, 32931; doi: 10.1038/srep32931 (2016).
